# Substandard and falsified antimicrobials in selected east African countries: A systematic review

**DOI:** 10.1371/journal.pone.0295956

**Published:** 2024-01-26

**Authors:** Addisu Afrassa Tegegne, Anbessa Bekele Feissa, Gemmechu Hasen Godena, Yesuneh Tefera, Hassen Kebede Hassen, Yildiz Ozalp, Sultan Suleman

**Affiliations:** 1 Pharmaceutical Sciences, Pharmaceutical Quality Assurance and Regulatory Affairs, University of Gondar, Gondar, Ethiopia; 2 Pharmaceutical Sciences, School of Pharmacy, Institute of Health Sciences, Jimma University, Jimma, Ethiopia; 3 Ethiopian Agricultural Authority, Veterinary Drug Quality Control and Inspection Directorate, Addis Ababa, Ethiopia; 4 Department of Pharmaceutical Technology, Near East University, Turkey; Institute of Tropical Medicine: Instituut voor Tropische Geneeskunde, BELGIUM

## Abstract

**Background:**

Globally, millions of people have been affected by fraudulent pharmaceutical products, particularly those in developing countries. Although the problem of falsified and substandard drugs is acknowledged, the extent of the issue is ever-changing, has a dynamic nature, and should be quantified and captured in a recent snapshot.

**Objective:**

This systematic review seeks to examine the data that can quantify and provide a current snapshot of the prevalence of SF antimicrobials in selected east Africa countries.

**Methods:**

Scientific studies on antimicrobial quality were searched in PubMed, Embase, Scopus, and Google Scholar from 2017 to February 2023. The search strategy focused on scientific articles published in peer-reviewed scientific journals written in English and the studies exclusively done in any of the selected countries of east Africa. The articles were carefully reviewed by two individuals for inclusion independently, first by title followed by abstract and the full-text retrieval. To minimize bias associated with the methodology used for data collection, the quality of the studies was assessed for quality according to the Medicine Quality Assessment Reporting Guidelines (MEDQUARG). The reporting of this systematic review was done following Preferred Reporting Items for Systematic Review and Meta-Analysis (PRISMA).

**Results:**

Fifteen studies that estimated the prevalence of poor-quality antimicrobial medicines in selected four east African countries were included. The overall percentage of samples of antimicrobials that failed at least one quality test was 22.6% (151/669) with each class’s prevalence of 17% in antibiotics (73/432), 24% in antimalarial (41/171), and 56% in anthelmintics (37/66). Quality control parameters of API content were the most commonly examined in the included studies, accounting for 14/15 (93%) studies. Fifty (33.1%) of the failing samples failed assay API- content determination, while 26.5% (n = 40) failed the visual inspection and packaging analysis; 19.2% (29) failed dissolution; 14% (n = 21) flawed hardness or friability; 4%(n = 6) failed uniformity, as well as 3.2% (n = 5) failed disintegration test of the quality control parameter.

**Conclusion:**

It was found that this review was general in these selected east African countries and was a catalyst for combating the menace of poor-quality medications that affect millions of lives.

## Introduction

Medicines are essential for better public health outcomes when they are of a quality that is intended to be unless they could negatively affect global health [1]. Aside from misuse and overuse of antimicrobials, poor-quality medicine also poses a threat to public health and can have devastating consequences for communities, where markets are at risk of being exploited by substandard and falsified pharmaceuticals [2]. Such products risk harming human health because they do not treat the intended disease or condition, and at worst, they can lead to death and morbidity [3]. Among the medicines that are susceptible to substandard or falsified products are antimicrobials used to treat infectious diseases [4]. According to WHO estimates, fewer than 30% of medicines regulatory authorities worldwide can ensure the safety of medicines, vaccines, and other health products. The WHO has certified five national organizations in Africa with maturity level three certification for medicines and imported vaccines, including Ghana, Nigeria, and Tanzania, as well as agencies in South Africa and Egypt that produce vaccines [5]. Many other countries are still working to create a stable, well-functioning, and integrated regulatory environment. Instabilities in regulatory structures can make it difficult to license manufacturers, ensure good manufacturing practices, and maintain quality control [6]. As a result, substandard and falsified medications could be manufactured and distributed.

Data from 2017, the World Health Organisation (WHO) reported that one in ten medicines, vaccines, and diagnostics fail quality testing in low-and middle-income countries [7]. It indicates that medicines do not meet quality specifications, are deliberately faked, and those not approved are circulating to heighten the risk of morbidity and mortality due to treatment failure, adverse drug reactions, and antimicrobial resistance [8, 9]. The antibiotics with the highest failures, which did not comply with standards, are among the critically important groups of antimicrobials in the WHO ranking [10, 11]. The availability of substandard or falsified antibiotics in developing countries, alongside poor surveillance of drug-resistant infections and clinical misuse [12], is one of the main drivers of antimicrobial resistance [13, 14].

A systematic review published in 2013 included prevalence studies in 25 low- and middle-income countries and found that the media prevalence of substandard and falsified medicines was 28.5% [15]. In other studies conducted after five years, the media prevalence has remained high at 25%, with little change since the previous study [16]. In comparison, a systematic review and meta-analysis study that included studies with 50 or more found that medicines were of poor quality, with a regional prevalence of 18.7% in Africa [8]. The perils of fraudulent pharmaceutical products have been affecting millions of people worldwide, particularly in developing countries. As of 2017, WHO data from Global Surveillance and Monitoring System estimate have accepted 1500 reports of substandard and falsified products since 2013, 42% of the reports come from the WHO African region [17]. Patients die every day in sub-Saharan African countries where 10% of the sampled medicines fall to be substandard or falsified from the lifesaving antimicrobials such as antimalarials, antibiotics, and antiretrovirals [18]. In its most recent threat assessment report, the United Nations Office on Drugs and Crime notes that almost half a million sub-Saharan Africans are killed by drug trafficking every year [19].

The actual trend in poor quality is a matter of considerable concern and a global effort is required to ensure a sustainable market system and ensure medicine quality [20]. The World Health Organization launched the Lomé Initiative, which criminalizes trafficking and imposes substantial penalties to address concerns about substandard and falsified medicines [21]. Nevertheless, Newton told The Lancet that substandard drugs should be regulated and not criminalized since most are caused by factory errors or degradation in the supply chain [22]. The Global Action Plan (GAP) of the WHO has resulted in the development of National Action Plan (NAP) across countries is among the strategies to combat substandard and falsified antimicrobials, but their implementation is often weak or stalling in low- and middle-income countries [23, 24]. NAPs in east African countries were mostly written in 2017 [25–27], including Ethiopia (2015) [28], which seeks to detect and respond to substandard and falsified medical products, and Eritrea (2021) [29], which seeks to improve quality assurance and post-market surveillance systems [30].

Understanding the extent of market penetration of poor-quality antimicrobials is important because it is associated with adverse health effects due to untreated infectious diseases or toxic ingredients, and accelerated emergence of drug resistance. Survival of more resistant pathogens can be enhanced, reducing consumers’ confidence in medicine, health care providers, and regulatory agencies [31]. Policymakers and medicine regulators have to be aware of the magnitude of poor-quality medicine incorporated into the market and make improvements in the country’s supply chain and drug post-market surveillance systems combined with efforts to provide equal access to high-quality products [32]. In this study, evidence that quantifies and captures a snapshot of the prevalence of SF antimicrobial medicines was reviewed. Health professionals, policymakers, and government regulators can be made aware of the burden of such a wicked problem and the need to formulate effective strategies to prevent and reduce the spread of substandard and falsified antimicrobials.

### Definitions

Substandard and falsified have been the WHO’s preferred terms since May 2017, substituting "substandard/spurious/falsely labeled/falsified/counterfeit". Falsified is used instead of counterfeit to avoid connotations associated with intellectual property. Generally, this refers to fake versions of real medications with false labels, packaging, and ingredients, whether they are innovators or generic products. Medical products that are "out of specification" are considered substandard, and are produced by legitimate manufacturers who fail to meet the quality standards that the manufacturer says they meet.

## Methods

### Databases and search strategy

The search strategy was created to find pertinent publications made between 2017 and February 2023. To locate all relevant terms, several literatures on the subject area were examined. Examples of the term use are; EMBASE: (counterfeit* OR fake OR falsified OR substandard) AND (drug* OR medicine* OR pharmaceutical* OR antimicrobial* OR antimalaria* OR antibiotic*) AND (’east Africa’/exp OR ’east Africa’); SCOPUS: TITLE-ABS-KEY (antimicrobial* OR antimalaria* OR antibiotic* OR pharmaceutical* OR drug* OR medicine*) AND (counterfeit* OR fake* OR spurious OR substandard OR falsified OR "poor quality*") AND ALL ("east Africa")); and Mesh terms from previous scientific papers were located and used in PubMed database search. Those terms were made in other combinations to search Google Scholar. In the search strategy, quotation marks were used to retrieve the exact phrase while an asterisk was applied as a wildcard. The quotation marks narrow the search by including only the exact phrase entered in the database search bar. An asterisk is used as a wildcard that can be applied to search for a single word or any variation of a word, such as plurals and conjugations. Furthermore, Boolean operators were implemented to increase the accuracy of the results.

### Inclusion and exclusion criteria

**Fig 1:**
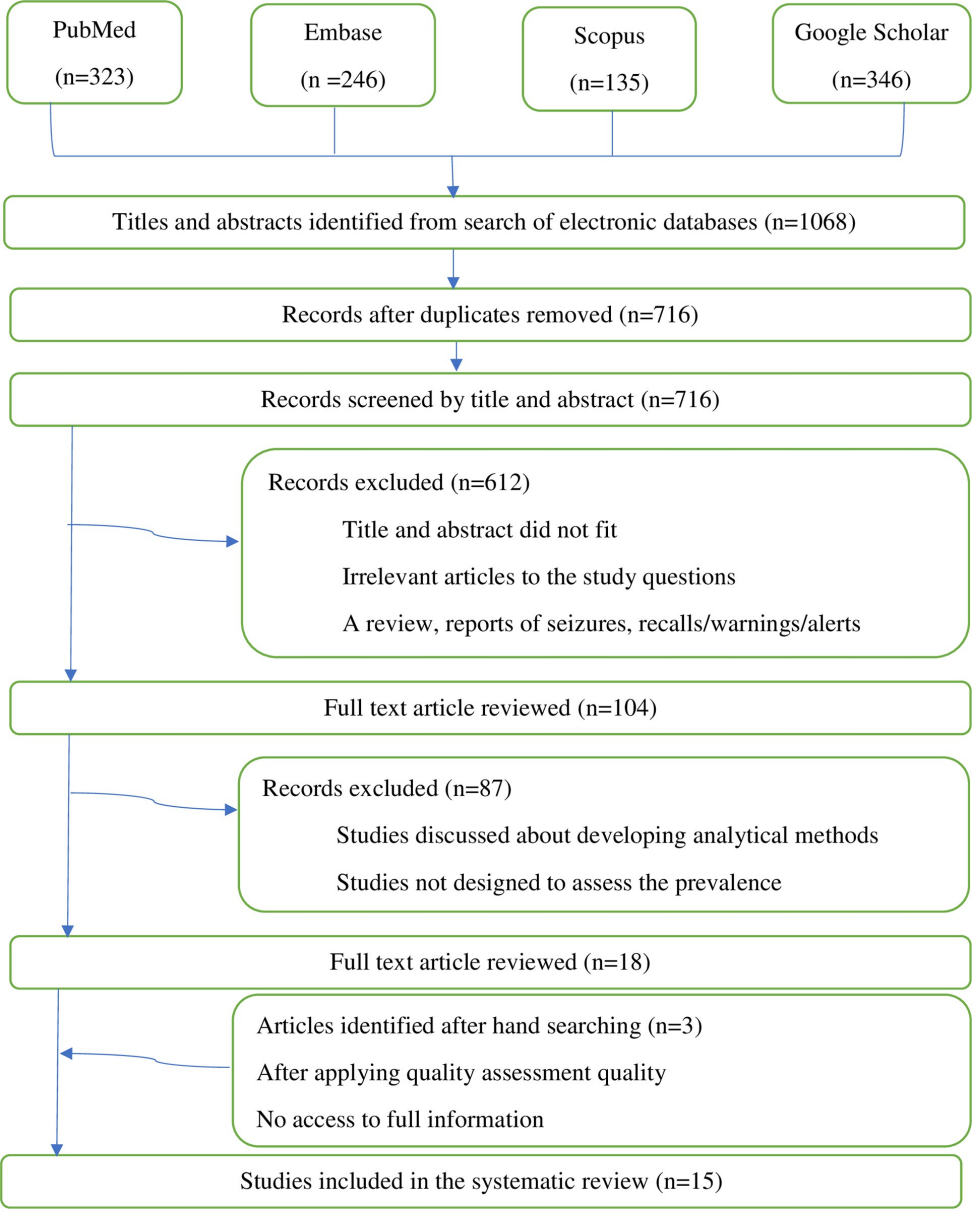
Flow diagram of the literature search strategy and review process.

The search strategy focused on papers published in peer-reviewed scientific journals written in English and studies exclusively done in any of the countries of east Africa (Burundi, Comoros, Djibouti, Ethiopia, Eritrea, Kenya, Madagascar, Mauritius, Mozambique, Réunion, Rwanda, Seychelles, Somalia, Somaliland, South Sudan, Tanzania, Uganda, Zambia, and Zimbabwe) [33]. Studies were excluded if they covered analytical techniques for the detection of substandard or falsified medicines or they took the form of a review, reports of seizures, recalls/warnings/alerts of antimicrobial medicines, and reports of adverse reactions where the quality of the medicine was suspected to be the cause. The most useful studies for this systematic review were those that surveyed the quality of antimicrobials medicines in one or more geographical locations referred to as prevalence surveys as they provide invaluable insight for the assessment, allowing for accurate and representative evaluation of the quality of medicines. Articles assessing the quality of herbal medicines, dietary supplements, and medicines used in animal health were excluded. The full search strategy and result flow diagram can be found in *Fig 1.*

### Selection of studies, data extraction, and reporting

All search results were imported to EndNote citation manager software, and duplicates were removed. Guided by a set of inclusion criteria, AA and AB scanned through all titles and abstracts and excluded articles that had irreverent titles. Before we retrieved the full text of the articles, the abstract and keywords of the remaining articles were evaluated and then we retrieved the full text. YT and GH retrieved and reviewed these to determine whether or not they met the criteria for inclusion in the study. The following data are extracted from each study independently by AA and YO based on a predesigned data extraction form: the type of drugs sampled, the sample size, the percentage of substandard or falsified medicines, the dosage forms included, the chemical analysis performed, and the location where it is done, the origin of the drugs, the issues associated with SF medicines, and the standard pharmacopeia used were all relevant factors to consider. To analyze the data generated after extraction from the various data sources, the data were entered into and analyzed descriptively with Microsoft Excel (2016). The reporting of this systematic review was done by following Preferred Reporting Items for Systematic Review and Meta-Analysis (PRISMA).

### Quality evaluation assessment

To minimize bias associated with the methodology used for data collection, the quality of the studies was evaluated. As part of the MEDQUARG checklist, 12 criteria were adopted to assess the quality of the methodology of each study included in the quality survey of the medicine. These requirements were listed in the MEDQUARG (Medicine Quality Assessment Reporting Guidelines) methodology section (*Table 1*). a checklist of elements to cover in reporting on surveys of the quality of medicines. A study must score between 6 and 12 according to the MEDQUARG checklist criteria to be considered methodologically sound and included in this systematic review.

**Table 1:** Quality assessment criteria.

1.	The time and location of the study clearly stated
2.	The definition of counterfeit or substandard medicines used mentioned
3.	Type of outlets sampled
4.	The sampling design and sample size calculation described
5.	Type and number of dosage units purchased per outlet
6.	Random sampling used
7.	Information on who collected the samples
8.	Packaging assessment performed
9.	Statistical analysis described
10.	The chemical analysis clearly described
11.	Details on method validation
12.	Chemical analysis performed blinded to packaging

## Result

### Selection and characteristics of eligible studies

**Fig 2:**
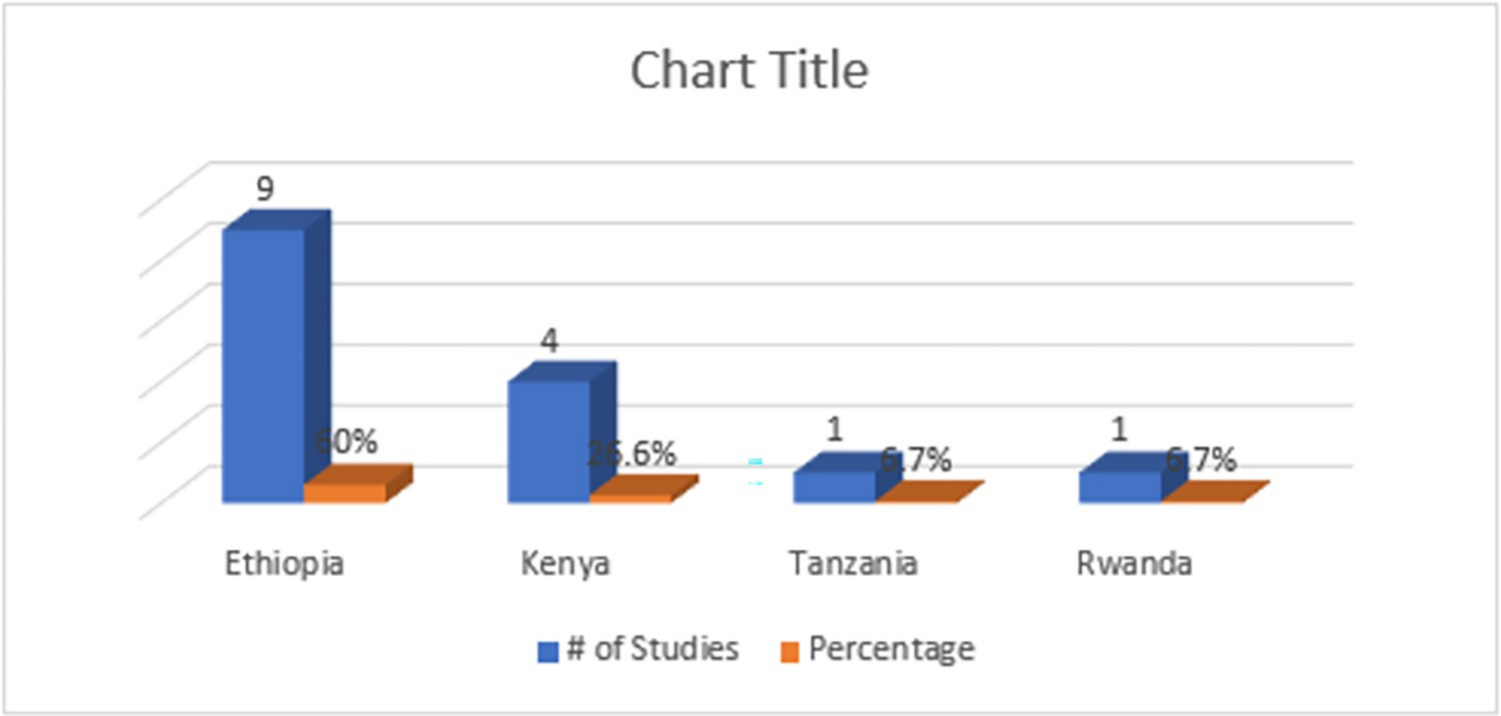
Location of selected studies in east Africa.

A review of studies in Ethiopia, Kenya, Rwanda and Tanzania was conducted; however, the remaining east African nations were not included due to article inclusion requirements. A flow diagram of literature searches and results can be seen in *Fig 1*. With the defined search strategy, a total of 1068 published articles (PubMed:323; Embase:246; Scopus: 135; Google Scholar: 346) were identified and their citations are exported to the endnote reference manager. After the removal of duplicates, 716 out of 1068 records gathered through electronic searches were screened by titles and abstracts. Of these, 104 full-text papers were retrieved to assess eligibility with 15 publications, published between 2017 and February 2023, included in the subsequent analysis after applying methodology quality assessment. Included publications are original research articles published in scientific journals comprising prevalence surveys and equivalence studies with a specified geographic region for which drug samples must be selected and tested. Approximately 10 of the articles included in this review are prevalence survey studies, which described prevalence surveys within the pharmaceutical supply chain using samples collected to assess the quality of circulating SF medicines. The remaining are equivalence studies that assessed the quality of different marketed brands of the same API(s) assuming that the results of the collected samples would represent the quality of the brand as a whole. Samples were collected from a wide range of outlets: medical stores department, wholesale pharmacies, public (e.g., health facilities, hospitals, and other official centers), private (pharmacies), and informal (street vendors and market stalls). Five out of 15 studies used random sampling in this review (33.3%) in which the sample was collected from outlets that were randomly selected in a defined area while the others are purposive, convenience or their sampling method is not specified clearly (*Table 2*). Within the review period, Ethiopia was found to have the highest number of prospective studies of SF medicines (9/15, 60%), followed by Kenya had the second highest number (4/15, 26.6%), Tanzania and Rwanda each accounted for (1/15, 6.7%) (*Fig 2*).

**Table 2:** Main characteristics of prevalence studies of antimicrobial quality in order of date of publications.

Reference	Countries	Journal/Publisher	Sampling method	Active Pharmaceutical Ingredients (API) Sampled	Brand name	Failed samples n/N (%)	The stated origin for products quality failures	Study types
Manani, 2017 [19]	Kenya	Scientia Pharmaceutica	Purposive	Clarithromycin	Klacid®, Klaricid®	0% (0/16)	None	Equivalence
Belew, 2018 [40]	Ethiopia	Science Direct	Random	Albendazole (ABZ), mebendazole (MBZ), praziquantel (PZQ)	Albezole®, Ovis®, Wormin®, Mebepharm, Wormin®, Thelmox®, Bermoxel®, Distocide®, Praziquantel	33.33% (3/9)	India, Cyprus,	Survey
Ndwigah, 2018 [43]	Kenya	BMC, Malaria Journal	Convenience	Artemether / luminescentrine, Quinine suspension, Dihydroartemesinin/piperaquine, Artesunate/mefloquine, Amodiaquine, Artemisinin/piperaquine, Artesunate/amodiaquine, Artemisinin/naphthoquine, Chloroquine, Artesunate injection, Artesunate suspension	Coartem®, AL-IPCA®Actm, Co-falcinum® AL Actm, Artefan, Lonart DS®, Cofantrine Forte®, Lumesoft Plus®, Lonart® suspension, Duo, cotexin®, P-Alaxin®, Ridmal®, Darte-Q®, Malacur, Artequick®, Asaq®, Artequin®, Arco®, Artesun®, Topquine®, Nelquine®, Falciquin®, Leoquin®, Remox®	0% (0/37)	None	Survey
Hambisa, 2019 [35]	Ethiopia	Dove Medical Press	Convenience	Norfloxacin	Negaflox, Norfen, Asnor, Norbek, Norcin, Uriflox, Gyrablock, Norflox, Trizolin,	22.22% (2/9)	Ethiopia, India	Equivalence
Mohammed, 2018 [41]	Ethiopia	Asian Journal of Pharmaceutical Research and Development	Unknown	Pantoprazole	Unspecified	0% (0/5)	None	Equivalence
Belew, 2019 [34]	Ethiopia	BMC, Malaria Journal	Unknown	artemether–lumefantrine	Coartem®, Artefan®, Artemine®	1.35% (1/74)	India	Survey
Desta, 2020 [36]	Ethiopia	SAGE, Journal of Generic Medicines	Random	Ciprofloxacin	Unspecified	0% (0/6)	None	Equivalence
Abuye, 2020 [39]	Ethiopia	Infection and Drug Resistance	Random	Chloroquine Phosphate and Quinine Sulfate	Quinil, Quinine	66.67% (40/60)	Ethiopia, India, Cyprus,	Survey
Koech, 2022 [45]	Kenya	BioMed Research International	Purposive	Amoxicillin and amoxicillin/clavulanic acid	Amoxil®, Augmentin®, Penamox, Elymox, Kamox, Kemoxyl, Moxacil, Amoximed, Unimox, Suprapen Rivamox, Amoxil Forte, Asmox, Moximed, Nesmox, Zeemox, Clavulin, Acinet, Clavam, Claxy, Amoklavin Curam, Clanoxy, Bactoclav, AmoxiClav Denk, Neoclav, Clav, Koact, Rapiclav	37.74% (20/53)	India, China, Egypt,	Survey
Abraham, 2021 [37]	Ethiopia	Advances in Pharmacological and Pharmaceutical Sciences	Convenience	Doxycycline hyclate	Teradoxin, Medomycin Doxylagap, Doxyleb Epadoxine, Doxycap Doxycad, Doxy denk Miraclin, Remycin	30% (3/10)	Italy Cyprus	Equivalence
Seitzer, 2021 [47]	Tanzania	PLoS Neglected Tropical Diseases	Random	Albendazole, Mebendazole and Praziquantel	Alben, Albi, Alzental, Anthel, Azentel, Benpham, Elyzole, Womiban, Zentel, Astazole, Mebrone, Natoa, Vermox, Wormnil, Bermoxel, Cesol, Distocide, Prazikant,	80% (32/40)	Tanzania, Cyprus, India, Kenya, South Korea	Survey
Irungu, 2021 [44]	Kenya	PLoS ONE	Purposive	Sulfamethoxazole and trimethoprim	Unspecified	29.2% (31/106)	Kenya, Egypt, India	Survey
Yirdaw, 2021 [38]	Ethiopia	Advances in Pharmacological and Pharmaceutical Sciences	Not specified	Ethambutol	Unspecified	0% (0/6)	None	Survey
Bizimana, 2022 [46]	Rwanda	MDPI, Antibiotics	Random	Amoxicillin, Co-trimoxazole, Cloxacillin, Erythromycin, Ciprofloxacin, Doxycycline Metronidazole, ampicillin, phenoxymethylpenicillin Ceftriaxone, Cefotaxime, Amoxicillin + Clavulanic acid	Lae-PenV, Unipen, Flagyl, Eflaron, Tricozole, Biotrocin, Erocin, Dox-Denk, Biodox, Doxil, Cipronat, Ciprolab, Cipro-Denk, Ciprobid, Aarciflox, Ceepro, Unitrim, Sulphatrim, Emtrim forte, Renetrim, Lecotrim, Astrim, Safraclav, AB-MET, ABAGYL, Tricozole, Metrogyl, Erythrox, Dawaclox, Cloxispa, Spamox, Texime, Clamoxyl, Moxacil, Ceftrimed, Cloxispa	8.19% (19/232)	Unspecified	Survey
Mekasha, 2023 [42]	Ethiopia	PLoS ONE	Convenience	Azithromycin	Swazi, Zithrotel, Zithrocin, Azitro, Azeescot, Zycin	0% (0/6)	None	Survey

The method used for drug analysis varied depending on the type of test, dosage form, and drug analyzed. In general, these samples were analyzed according to pharmacopeia specifications (*Table 3*). Eight out of the 15 included studies in this review did not mention the information on the individual who collected the samples.

**Table 3:** General description of the prevalence of falsified and substandard medicines.

Country (reference)	Drugs (n = number of various products tested)	Setting/outlet	Formulation studied	Labeled origin	Method of Testing/location	Characteristics of Falsified/substandard drugs	%(substandard/falsified)	Standard pharmacopeia
Ethiopia [34]	Antimalarial drugs, fixed-dose combination artemether (ART)/lumefantrine (LUM) (n = 74)	Public facilities	Tablets	Ethiopia, China, India, USA	DAD-UV Spectroscopy, TLC, HPLC method, visually inspected for physical characteristics of tablets/ Jimma University Laboratory of Drug Quality (JuLaDQ), Ethiopia	An excessive quantity of active ingredient quantity	1.35% (1/74 substandard)	International Pharmacopoeia, European Pharmacopoeia
Ethiopia [35]	Antibiotics, norfloxacin (n = 9)	Hospital/ Health centers, Private pharmacy	Tablets	Ethiopia, India, South Korea, Cyprus	HPLC, dissolution, friability, tablet hardness, mass uniformity/ Jimma University drug quality laboratory (JuLaDQ), Ethiopia	Dissolution test failure	22.22% (2/9 substandard/falsified)	USPNF
Ethiopia [36]	Antibiotics, ciprofloxacin (n = 6)	Private retail outlets	Tablets	Ethiopia, India, South Korea, Cyprus Germany	HPLC, disintegration, dissolution—UV-vis spectrophotometer, mass uniformity	None	0% (0/6 substandard/falsified)	USP/NF
Ethiopia [37]	Antibiotics, Doxycycline Hyclate (n = 10)	Private retail outlets	Tablets and Capsules	Ethiopia, India, South Korea, Cyprus, Germany, Switzerland, Italy	API content and identification—HPLC, dissolution, friability, tablet hardness, visual inspection, mass uniformity/ Jimma University drug quality laboratory (JuLaDQ), Ethiopia	failure of the hardness and friability tests failure	30% (3/10 substandard)	International Pharmacopeia, United State Pharmacopeia
Ethiopia [38]	Antibiotic, Ethambutol HCL (n = 6)	Governmental health facilities	Tablets	Unspecified	API content -HPLC, dissolution, weight variation and friability test, visual inspection/ Jimma University drug quality laboratory (JuLaDQ), Ethiopia	None	0% (0/6 substandard/falsified)	USP/NF
Ethiopia [39]	Antimalarials, Chloroquine Phosphate and Quinine Sulfate Tablets (n = 60)	Drug stores (pharmacy, drug). store, and drug vendor	Tablets	Ethiopia, India, Cyprus	HPLC -identification and assay/content, dissolution, dosage uniformity—UV—visible spectrophotometer, hardness, friability tests, visual inspection and organoleptic property checking/ Jimma University drug quality laboratory (JuLaDQ), Ethiopia	Failed to comply with the Pharmacopeial quality standards for visual inspection, hardness, and weight variation tests,	66.67% (40/60 substandard)	USP-2015
Ethiopia [40]	Anthelminthic medicines; albendazole (ABZ), mebendazole (MBZ), praziquantel (PZQ) (n = 9)	Retail pharmacies	Tablets	India, Cyprus	Amount of API -HPLC, disintegration and dissolution test, mass uniformity, Visual inspection/ Jimma University drug quality laboratory (JuLaDQ), Ethiopia	failing the United States Pharmacopoeia (USP) dissolution specification limit.	33.33% (3/9 substandard)	European Pharmacopoeia 2014, United States Pharmacopoeia method (USP, 2015)
Ethiopia [41]	Antibiotic, Pantoprazole Sodium Enteric Coated Tablets (n = 5)	Different retail outlets	Tablets	Germany, Turkey, India, Saudi Arabia, Slovenia	Thickness- sliding caliper scale, crushing strength- the hardness tester, Weight uniformity of dosage units, disintegration time/Addis Ababa, Ethiopia	None	0% (0/5 substandard/falsified)	USP/NF (2013)
Ethiopia [42]	Antibiotic, Azithromycin (n = 6)	Drug retail outlets	Tablets	India, Ethiopia, Turkey, Greece	HPLC; identity, assay tests, Hardness, friability, weight variation, disintegration, and visual inspection for physical characteristics/ Ethiopian Pharmaceutical Company Drug Quality Control Laboratory (EPHARM)	None	0% (0/6 substandard/falsified)	United States Pharmacopeia
Kenya [19]	Antibiotics, Clarithromycin (n = 16)	Private retail outlets	Suspensions, Tablets, Capsules	South Africa, UK	HPLC, dissolution test/Nairobi County, Kenya	None	0% (0/16 substandard/falsified)	USP (2014)
Kenya [43]	Antimalarial drugs, artemisinin combination therapy (n = 37)	Public and private outlets	Tablets	Local and imported	HPLC, UV spectroscopy / Drugs and Analysis Research unit, University of Nairobi, Nairobi, Kenya	None	0% (0/37 substandard/falsified)	United States Pharmacopoeia (USP-2018)
Kenya [44]	Antibiotic, Trimethoprim/sulfamethoxazole (n = 106)	Private retail pharmacies	Suspension	Kenya, India and Egypt.	Determination of API content by HPLC, Thermo Scientific pH Metre, Packaging, and labeling inspection/ Kenya Medical Research Institute, Centre for Traditional Medicine and Drug Research, Kenya	Excessive and insufficient active ingredient quantity, Visual inspection failure, failure to comply with USP upper limit pH specifications	29.2% (31/106 substandard)	USP (2017)
Kenya [45]	Antibiotics, Amoxicillin (n = 53)	Retail and private hospital pharmacies	tablets, Capsules, powders for oral suspension, Suspension,	India, UAE, Canada, UK, France, Jordan, China, Kenya, Mexico	HPLC, API Determination, Dissolution, determination of Uniformity of Weight / Kenyatta University, Kenya.	Inadequate active ingredient quantity, dissolution test and weight uniformity failure	37.74% (20/53 substandard or falsified)	USP-NF
Rwanda [46]	12 Most-Used Antibiotics; amoxicillin, co-trimoxazole, Cloxacillin, Erythromycin, Ciprofloxacin, Doxycycline, Metronidazole, Phenoxymethylpenicillin, Ampicillin, Ceftriaxone, Cefotaxime, Amoxicillin + Clavulanic acid (n = 232)	Private retail pharmacies	tablets, Capsules, powders for oral suspension, Suspension, Powders for Injection	Kenya, India, China, France, Turkey, Germany, Italy, Uganda, Morocco, Senegal	Visual inspection with International Pharmaceutical Federation (FIP) tool, and assay tests with HPLC-DAD/ Division of Quality Control Laboratory, Rwanda Food and Drugs Authority, Kigali, Rwanda.	Inadequate active ingredient quantity	8.19% (19/232 substandard)	United States Pharmacopoeia 2018
Tanzania [47]	Antihelminth; Albendazole, Mebendazole and Praziquantel (n = 40)	Private and Hospital Pharmacy	Suspension, tablets	Tanzania, India, South Korea, Kenya, South Africa, France, Cyprus, Mexico	HPLC, API Determination, Dissolution, Mass uniformity, dissolution test, TLC identity, Disintegration times, visual inspections/ Various locations	Insufficient quantity of active quantity of ingredients, disintegration, and dissolution test, not complying with the packaging information requirements	80% (32/40 substandard)	USP-NF International Pharmacopoeia

### Prevalence of substandard or falsified medicines

There were 669 samples collected from four countries representing 28 different types of API or API combinations. Of all samples, 22.6% (151/669) failed at least one quality control parameter test. The sample size ranged from 5 to 232 samples per study with a mean of 44.6 samples. The median prevalence of substandard/falsified medicines (at least one quality test failing) was 8.19% (range 0%–80%). Falsified or substandard medicines accounted for 151 samples, of which 73 (48%) were antibiotics followed by antimalarials and anthelmintics, which account for 41 (27%), and 37 (25%), respectively. Randomly selected samples had the highest SF (SF = 27%, 94/347), followed by convenience or purposive selection samples (23.6%, 56/237) and unstated/unclear sampling strategies (SF = 1.2%, 1/85).

### Quality control parameters and stated issues of substandard/falsified medicines

**Fig 3:**
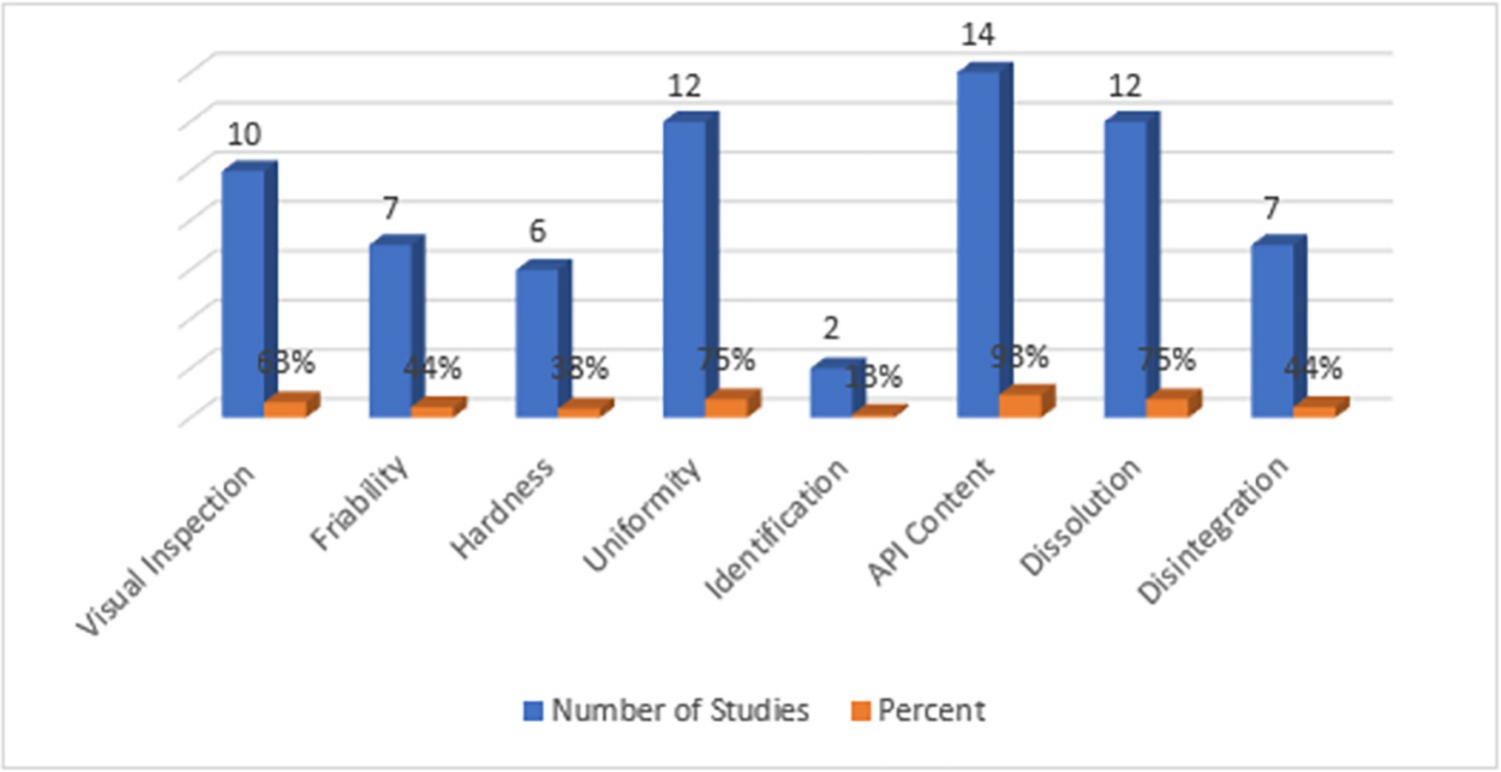
The type and proportion of quality control parameters addressed in the included studies.

A minimum of two quality control parameters were evaluated in each study: packaging and labeling of pharmaceutical inspections, hardness, friability, weight variation, disintegration, dissolution, identification, and assay/percentage purity test. *Table 3* shows the most common problems reported by these studies concerning substandard or falsified antimicrobial drugs. The determination of API content was the most examined quality control parameter most examined, being determined by all of the studies except one [41] as shown in *Fig 3.* Most of the studies 13/15 (86.7%) test chemical analysis for determination of the assay and the identification tests by HPLC or TLC. Visual inspection was done in 10/15 (66.7%) of the included studies. Moreover, the physical analysis disintegration and dissolution tests were performed, in 7/15 (46.7%) and 11/15 (73.3%) of the studies respectively. Fifty (33.1%) of the failing samples failed assay API- content determination, while 26.5% (n = 40) failed the visual inspection and packaging analysis; 19.2% (29) failed dissolution; 14% (n = 21) flawed hardness or friability; 4%(n = 6) failed uniformity, as well as 3.2% (n = 5) failed disintegration test of the quality control parameter.

### Characteristics of the drug and therapeutic classes

**Fig 4:**
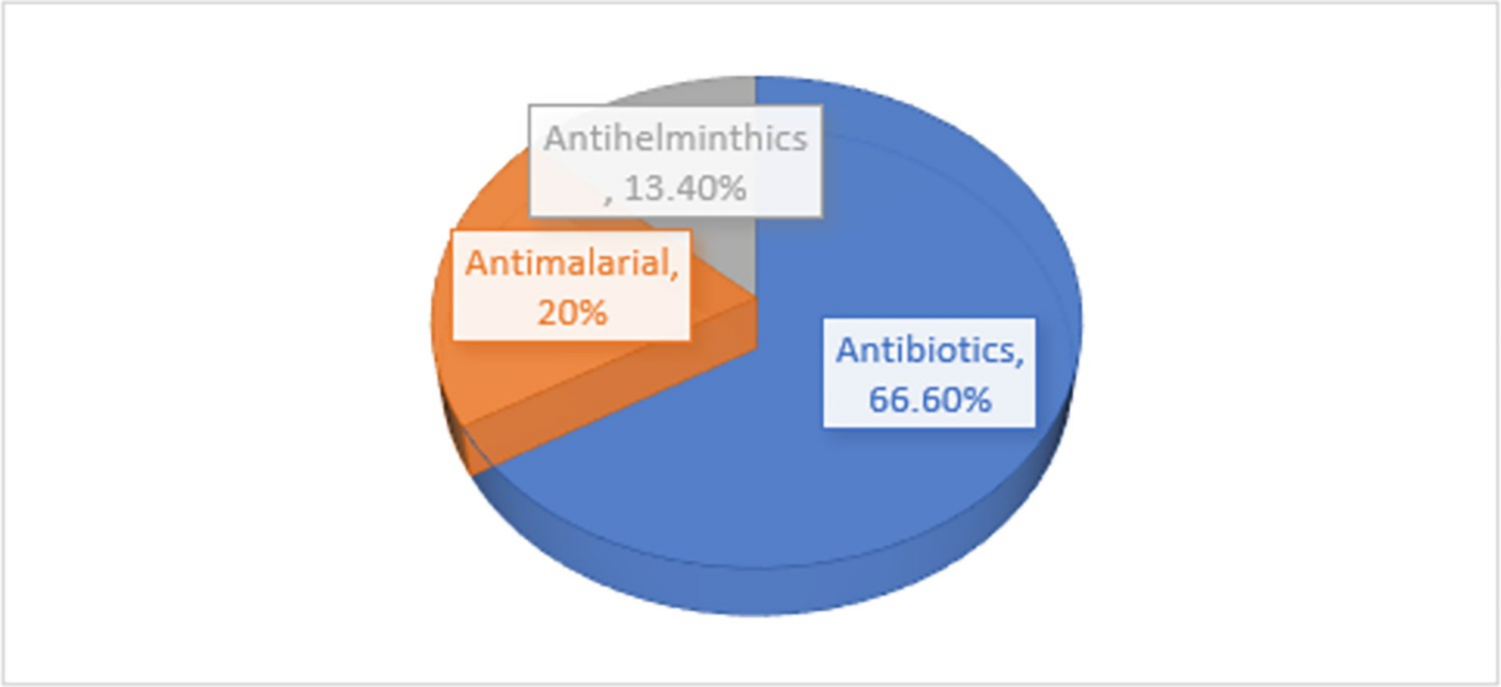
The proportion of antimicrobials categories examined by the included studies.

In total, 669 APIs or combinations of APIs originating from different countries were tested for quality, which are of various antimicrobial therapeutic categories. Among the studies included, the majority of 10/15 (66.6%) focused on the antibiotics class of drugs that treat infectious diseases. Antimalarial and anthelmintics medicines were examined in 3/15 (20%), and 2/15(13.4%) of the located studies, respectively (*Fig 4*). Five of the 15 studies examined the quality of multiple drugs belonging to the same therapeutic class. 3/15 (20%) of the included published articles assessed a fixed-dose antimicrobial combination of two or more active pharmaceutical ingredients, while the remaining studies evaluated a single active pharmaceutical ingredient.

From the samples collected in each antimicrobial category, the failed antibiotic sample was 16.9%, of which co-trimoxazole and ceftriaxone had 25% failure followed by amoxicillin, norfloxacin, and ampicillin, 23%, 22%, and 20% respectively. Chloroquine failed with 73% of the collected sample and followed by quinine at 32%. Anthelmintics have been identified as failed, including mebendazole (79%), praziquantel (75%), and albendazole (65%) (*Table 4*).

**Table 4:** Class of each antimicrobial along with percentage failure.

Class of Antimicrobial	Name of API	Failed Sample	Total	Percent (%)
Antibiotics	Phenoxymethylpenicillin	0	7	0
	Ceftriaxone	1	4	25
	Ciprofloxacin	0	26	0
	Doxycycline	3	29	10
	Cloxacillin	2	31	6
	Clarithromycin	0	16	0
	Pantoprazole	0	5	0
	Azithromycin	0	6	0
	Erythromycin	1	25	4
	Norfloxacin	2	9	22
	Co-trimoxazole	36	143	25
	Ethambutol	0	6	0
	Amoxicillin	27	117	23
	Cefotaxime	0	3	0
	Ampicillin	1	5	20
	Total	73	432	16.9
Antimalarial	Artesunate/ Amodiaquine	0	1	0
	artemether/ lumefantrine	1	92	1
	Quinine	7	22	32
	Chloroquine	33	45	73
	Dihydroartemisinin/piperaquine	0	4	0
	Artesunate/mefloquine	0	2	0
	Artesunate	0	2	0
	Amodiaquine	0	1	0
	Artemisinin/piperaquine	0	1	0
	Artemisinin/naphthoquinone	0	1	0
	Total	41	171	23.9
Anthelmintics	Metronidazole	2	17	12
	Albendazole	15	23	65
	Praziquantel	9	12	75
	Mebendazole	11	14	79
	Total	37	66	56
Class of antimicrobials	Total failed samples (percentage)	Total samples tested (percentage)		
Antibiotics	73(48.3)	432(64.6)		
Antimalarial	41(27.2)	171(25.6)		
Anthelmintics	37(24.5)	66(9.8)		

## Discussion

This systematic review aimed to consolidate the current data on SF medicines reported in scientific studies. Data on SF antimicrobial medicines were extracted and reviewed from resources, available in the journal databases, from 2017 to February 2023. These are surveys and equivalence studies focused on the quality of antimicrobials and samples were collected in four different countries of east Africa. The overall SF was 22.6% of a total of 669 collected samples. Antibiotics were responsible for 48.3% of the failed samples, antimalarials for 27.2%, and anthelmintics for 24.5%. Similar to the previous study conducted globally, API content defects are the most frequently encountered quality parameter [11]. There was strong evidence in the results of samples with an inadequate amount of active ingredients (26.7% of studies), excessive active ingredients (13.3%), and dissolution failure (13.3%). A decade ago, a study conducted to explore the global evidence of poor-quality medicines identified 15 studies, 93% of the studies found inadequate amounts of API, and 33% found dissolution failure [15]. Patients could take medicine at a low or high dose as a result of these defects, resulting in treatment failure and unwanted toxic effects [48]. In terms of the quality of included studies, a large proportion were found to have poor methodological quality. From the studies that met quality inclusion criteria, only 2 of 15 studies followed and mentioned the MEDQUARG. Non-random sampling (convenience or purposive sampling) was often preferred and investigators collected samples haphazardly based on what outlets and wards could easily access them [49]. Despite its convenience and affordability, this method may be biased and may not be representative of the target area [50]. An estimate of a sample size has to be made and a random sample is chosen from a complete list of the area resulting in more reliable and accurate measures [49, 50].

There were nearly 17% of substandard or falsified antibiotics in the collected sample of antibiotics, compared to 18.7% of SF antibiotics reported in the previous studies [51]. Ceftriaxone, ampicillin, and amoxicillin-clavulanic acid are among the substandard and falsified that the WHO prioritize as being of critical importance antimicrobials [10]. In Africa, these substandard and falsified antibiotics are among the most commonly prescribed antibiotics. A systematic review of these antibiotics revealed the prevalence of ceftriaxone (7.4–51.7%), metronidazole (14.6–44.8%), gentamicin (6.6–22.3%), ampicillin (6.0–29.2%), ciprofloxacin (7.8–17.4%), and amoxicillin-clavulanate (8.8–13.4%) [52]. From the collected antibiotic sample, co-trimoxazole and ceftriaxone failed equally at 25%, followed by amoxicillin, norfloxacin, and ampicillin at 23%, 22%, and 20%, respectively. Due to their poor quality, these antibiotics are more likely to cause morbidity and death if left untreated, since they do not treat the diseases or conditions and may accumulate resistance over time [10]. The other group of antimicrobials was antimalarials which were identified with the failure of 24% of at least one quality control parameter. Antimalarial drugs have been commonly targeted, and up to 90% of antimalarial drugs were found to be of low quality in a WHO study in Africa [53]. Chloroquine was the highest substandard or falsified antimalarial drug with a percentage failure of 73%, followed by quinine, which was 32%. Over 90% of the cases of uncomplicated malaria in sub-Saharan African countries are caused by Plasmodium falciparum parasite resistance to chloroquine, the medicine which was found to be the highest falsified and substandard in this review [54]. Regarding the poor anthelmintics identified as substandard or falsified, mebendazole (79%), praziquantel (75%), and albendazole (65%), which were also reported in the previous review as not having active ingredients, increased impurities or altered labeling during visual inspections [55].

This data has been demonstrated that low-quality medicines in such countries are of considerable prevalent which are likely consumed by a significant number of people, resulting in higher mortality, morbidity, and antimicrobial resistance. Furthermore, the data inform pharmacists of the magnitude of SF, as it is a significant step towards tackling it, and they have to work diligently to spread the word about the issues among healthcare professionals, patients, and the general public about the risks of buying them illegally. In particular, governments should take significant steps to improve pharmaceutical governance and to strengthen technical capacity of regulatory laboratories, particularly in poor and rural communities. It is hoped that approaches such as the Lomé Initiative will lead governments to invest more in people and infrastructure in order to enhance regulatory capacity to counter SF medicines and provide better access to good quality antimicrobials [21, 22]. There are currently NAPs in east African countries, but a longer-term funding plan is needed, as well as the sustained commitment of national leaders and actors at the country level [23, 30]. Enhancing the participation of nations in WHO’s global surveillance and monitoring system and member States’ mechanisms concerning substandard and falsified medical products is also an important component of their national action plan for preventing, detecting, and responding to substandard and falsified medical products [7].

Limitations are searches were carried out only in English and original published papers were identified, not information from repositories, MRA websites, and other websites interested in the quality of medicines. In spite of our attempts to include data from a wide range of east African countries, we were unable to find relevant literature that met the specified criteria. The study focused on the data available from these four selected east African countries, as a consequence. The majority of studies used nonrandom sampling and a small sample size, which increased bias risks. As reflected in the low MEDQUARG scores, prevalence surveys were also poorly reported. Even though most of the studies completed after the publication of the MEDQUARGS in 2009 centered on human medicines, only two of the prevalence studies followed the guidelines for reporting their results.

## Conclusion

Our study found that 151 out of 669 single and combination antimicrobials tested were substandard or falsified across the selected east African countries: Ethiopia, Kenya, Rwanda, and Tanzania. The antibiotics were taking 64.6 percent of the total collected samples of which 17 percent were substandard or falsified medicines. Our finding suggests that organizations should evaluate and focus efforts on any measure to combat such proliferation of substandard and falsified antimicrobials, including strengthening their legal, financial, and purchasing frameworks, with these prevalence data. It is crucial to conduct well-designed prevalence studies that provide detailed methods in the future to gain a deeper understanding and improve generalizability.

## Supporting information

S1 Checklist (DOCX)

S1 File (DOCX)
