# Supplementary material for: Substandard and falsified antimicrobials in selected east African countries: A systematic review
Source: PLoS One. 2024 Jan 26;19(1):e0295956. doi: 10.1371/journal.pone.0295956 (PMC10817106; doi:10.1371/journal.pone.0295956)
Supplement: S1 Checklist — (DOCX) [file pone.0295956.s001.docx]

| **Section and Topic** | **Item #** | **Checklist item** | **Location where item is reported** |
| --- | --- | --- | --- |
| **TITLE** | | |  |
| Title | 1 | **Substandard and Falsified Antimicrobial in East Africa: A Systematic Review** | 1 |
| **ABSTRACT** | | | 2 |
| Abstract | 2 | Objectives, methods and results from analysis of primary data source and conclusion from findings is described | 2 |
| **INTRODUCTION** | | | 3 |
| Rationale | 3 | Assessment of quality of marketed products would have immense value to identify for gaps in quality assessment and product quality regulation |  |
| Objectives | 4 | Examine the data that can quantify and provide a current snapshot of the prevalence of SF medical products in East Africa from available primary data source |  |
| **METHODS** | | | 4 |
| Eligibility criteria | 5 | Scientific articles published in peer-reviewed scientific journals written in English and the studies exclusively done in any of the countries of east Africa | 4 |
| Information sources | 6 | All published journals available online through search engines extracted via the use of selected key words and search string | 5 |
| Search strategy | 7 | Using specific criteria, all available primary data sources are gathered and filtered | 5-6 |
| Selection process | 8 | Language of study presentation, study area coverage, primary study finding on quality assessment | 6 |
| Data collection process | 9 | Scientific articles searched and collected in PubMed, Embase, Scopus, and Google Scholar from the period of 2017 to February 2023 | 6 |
| Data items | 10a | Quality parameters from individual findings, reference standards used, type and category of each study drug, dosage form | 7 |
|  | 10b | No human subject inclusion | na |
| Study risk of bias assessment | 11 | Bias can only arise from lack of access to full information | na |
| Effect measures | 12 | Results are described in descriptive statistics | 7 |
| Reporting bias assessment | 14 | NA |  |
| Certainty assessment | 15 | A repeated search by different individuals minimizes missing data and bias |  |
| **RESULTS** | | | 8 |
| Study selection | 16a | 104 full texts publications were retrieved | 10 |
|  | 16b | 15 research articles were encountered after refinement with specific criteria | 10 |
| Study characteristics | 17 | Only publications that are indexed, containing quality assessment findings on marketed drugs in English language and samples from one of the east African markets were taken and considered. | 11 |
| Risk of bias in studies | 18 | Some primary data may be missed due to inaccessibility to full articles | na |
| Results of individual studies | 19 | There is a possibility that individual data points are not representative of the whole of its kind | na |
| Results of syntheses | 20a | There may be minor bias in percentages in descriptive statistics |  |
|  | 20b | Descriptive statistics used | 14 |
|  | 20c | It is possible for individual test results to be heterogeneous as a result of changes in method and sample size | na |
| Reporting biases | 21 | Individual data may be missed but what is found and demonstrated do have minimal bias | 14 |
| Certainty of evidence | 22 | All individual sources of primary data are extracted from well reviewed and indexed publication | 11-12 |
| **DISCUSSION** | | | 16 |
| Discussion | 23a | Results are compared with findings from other countries | 16-17 |
|  | 23b | Solid dosage formulations quality studies are encountered more in publications | 17 |
|  | 23c | NA |  |
|  | 23d | Organizations should evaluate and focus efforts on any measure to combat such proliferation of substandard and falsified antimicrobials | 18 |
| **OTHER INFORMATION** | | |  |
| Registration and protocol | 24a | NA |  |
|  | 24b | NA |  |
|  | 24c | NA |  |
| Support | 25 | NA |  |
| Competing interests | 26 | No competing interest among authors | 18 |
| Availability of data, code and other materials | 27 | template data collection forms; data extracted from included studies; data used for all analyses; any other materials used in the review. |  |
