# Supplementary material for: Substandard and falsified antimicrobials in selected east African countries: A systematic review
Source: PLoS One. 2024 Jan 26;19(1):e0295956. doi: 10.1371/journal.pone.0295956 (PMC10817106; doi:10.1371/journal.pone.0295956)
Supplement: S1 File — (DOCX) [file pone.0295956.s002.docx]

| **No.** | **Quality assessment criteria** | **Belew et al (2019)[1]** | **Desta, Haile et al (2020)[2]** | **Abuye, Habtamu, et al (2020)[3]** | **Yirdaw et al (2021)[4]** | **Abraham W et al (2021)[5]** | **Hambisa, Solomon et al (2019)[6]** | **Nigatu et al**  **(2019)[7]** | **Belew, Sileshi, et al (2018)[8]** | **Mekasha, Yet al (2023)[9]** |
| --- | --- | --- | --- | --- | --- | --- | --- | --- | --- | --- |
| 1 | Timing and location of study clearly stated. | $\surd$ | $\surd$ | $\surd$ | $\surd$ | $\surd$ | $\surd$ | $\surd$ | $\surd$ | $\surd$ |
| 2 | Definition of counterfeit or substandard  medicines used mentioned. | $\times$ | $\times$ | $\times$ | $\surd$ | $\times$ | $\times$ | $\times$ | $\times$ | $\times$ |
| 3 | Type of outlets sampled. | $\surd$ | $\surd$ | $\surd$ | $\surd$ | $\surd$ | $\surd$ | $\times$ | $\surd$ | $\surd$ |
| 4 | Sampling design and sample size calculation described | $\times$ | $\times$ | $\surd$ | $\times$ | $\times$ | $\surd$ | $\times$ | $\surd$ | $\surd$ |
| 5 | Type and number of dosage units purchased per outlet | $\surd$ | $\surd$ | $\surd$ | $\surd$ | $\surd$ | $\surd$ | $\surd$ | $\surd$ | $\surd$ |
| 6 | Random sampling used. | $\surd$ | $\surd$ | $\surd$ | $\surd$ | $\surd$ | $\times$ | $\times$ | $\surd$ | $\times$ |
| 7 | Information on who collected the samples | $\surd$ | $\surd$ | $\surd$ | $\times$ | $\surd$ | $\surd$ | $\times$ | $\times$ | $\surd$ |
| 8 | Packaging assessment performed. | $\surd$ | $\times$ | $\surd$ | $\surd$ | $\surd$ | $\times$ | $\times$ | $\surd$ | $\surd$ |
| 9 | Statistical analysis described. | $\surd$ | $\surd$ | $\surd$ | $\surd$ | $\surd$ | $\surd$ | $\surd$ | $\surd$ | $\surd$ |
| 10 | Chemical analysis clearly described | $\surd$ | $\surd$ | $\surd$ | $\surd$ | $\surd$ | $\surd$ | $\surd$ | $\surd$ | $\surd$ |
| 11 | Details on method validation | $\surd$ | $\surd$ | $\surd$ | $\surd$ | $\times$ | $\surd$ | $\surd$ | $\surd$ | $\surd$ |
| 12 | Chemical analysis performed blinded to packaging | $\surd$ | $\surd$ | $\times$ | $\surd$ | $\times$ | $\surd$ | $\surd$ | $\surd$ | $\surd$ |
|  | Score | 10/12 | 9/12 | 9/12 | 10/12 | 9/12 | 9/12 | 6/12 | 10/12 | 10/12 |

| **No.** | **Quality assessment criteria** | **Koech, Lilian C., et al(2020)[10]** | **Manani, Rebecca et al (2020)[11]** | **Irungu, Beatrice Njeri, et al (2021)[12]** | **Ndwigah, S et al**  **(2018)[13]** | **Sophia Mzirayet al (2021)[14]** | **Thomas Bizimana et al(2022)[15]** | **Seitzer M et al (2021)[16]** |
| --- | --- | --- | --- | --- | --- | --- | --- | --- |
| **1** | Timing and location of study clearly stated. | $\surd$ | $\surd$ | $\surd$ | $\surd$ | $\surd$ | $\surd$ | $\surd$ |
| **2** | Definition of counterfeit or substandard  medicines used mentioned. | $\times$ | $\times$ | $\times$ | $\times$ | $\times$ | $\times$ | $\times$ |
| **3** | Type of outlets sampled. | $\surd$ | $\surd$ | $\surd$ | $\surd$ | $\surd$ | $\surd$ | $\times$ |
| **4** | Sampling design and sample size calculation described | $\surd$ | $\surd$ | $\surd$ | $\times$ | $\surd$ | $\surd$ | $\times$ |
| **5** | Type and number of dosage units purchased per outlet | $\surd$ | $\surd$ | $\surd$ | $\surd$ | $\surd$ | $\surd$ | $\surd$ |
| **6** | Random sampling used. | $\times$ | $\times$ | $\times$ | $\times$ | $\times$ | $\surd$ | $\times$ |
| **7** | Information on who collected the samples | $\times$ | $\times$ | $\times$ | $\times$ | $\surd$ | $\times$ | $\surd$ |
| **8** | Packaging assessment performed. | $\times$ | $\times$ | $\times$ | $\surd$ | $\surd$ | $\surd$ | $\surd$ |
| **9** | Statistical analysis described. | $\surd$ | $\surd$ | $\surd$ | $\times$ | $\surd$ | $\surd$ | $\times$ |
| **10** | Chemical analysis clearly described | $\surd$ | $\surd$ | $\surd$ | $\surd$ | $\surd$ | $\surd$ | $\surd$ |
| **11** | Details on method validation | $\surd$ | $\surd$ | $\surd$ | $\surd$ | $\surd$ | $\surd$ | $\surd$ |
| **12** | Chemical analysis performed blinded to packaging | $\surd$ | $\surd$ | $\surd$ | $\times$ | $\surd$ | $\surd$ | $\surd$ |
|  | Score | 8/12 | 8/12 | 8/12 | 6/12 | 10/12 | 10/12 | 8/12 |

1. Belew, S., et al., *Quality of fixed dose artemether/lumefantrine products in Jimma Zone, Ethiopia.* Malar J, 2019. **18**(1): p. 236.

2. Desta, H.K. and T.T. Teklehaimanot, *In vitro quality evaluation of generic ciprofloxacin tablets available in community pharmacies of Dessie town, Northeast Ethiopia.* Journal of Generic Medicines, 2020.

3. Abuye, H., et al., *Physicochemical quality assessment of antimalarial medicines: chloroquine phosphate and quinine sulfate tablets from drug retail outlets of South-West Ethiopia.* 2020: p. 691-701.

4. Yirdaw, M., B. Umeta, and Y. Mokennen, *Quality Evaluation of Ethambutol Hydrochloride Tablet Batches Available in Governmental Health Facilities of Jimma Town, Southwest Ethiopia.* Advances in Pharmacological and Pharmaceutical Sciences, 2021. **2021**.

5. Abraham, W., et al., *In vitro comparative quality assessment of different brands of doxycycline hyclate finished dosage forms: capsule and tablet in jimma town, south-west ethiopia.* 2021. **2021**: p. 1-10.

6. Hambisa, S., S. Belew, and S. Suleman, *In vitro comparative quality assessment of different brands of norfloxacin tablets available in Jimma, Southwest Ethiopia.* Drug Des Devel Ther, 2019. **13**: p. 1241-1249.

7. Nigatu, M., et al., *In Vitro Comparative Study of Different Brands of Pantoprazole Sodium Enteric Coated Tablets Marketed In Addis Ababa, Ethiopia.* 2019. **7**(1): p. 13-18.

8. Belew, S., et al., *Quality of anthelminthic medicines available in Jimma Ethiopia.* Acta Trop, 2018. **177**: p. 157-163.

9. Mekasha, Y.T., et al., *Quality evaluation of the Azithromycin tablets commonly marketed in Adama, and Modjo towns, Oromia Regional State, Ethiopia.* PLoS One, 2023. **18**(3): p. e0282156.

10. Koech, L.C., et al., *Quality and Brands of Amoxicillin Formulations in Nairobi, Kenya.* BioMed Research International, 2020. **2020**.

11. Manani, R.O., K.O. Abuga, and H.K.J.S.P. Chepkwony, *Pharmaceutical equivalence of clarithromycin oral dosage forms marketed in Nairobi county, Kenya.* 2017. **85**(2): p. 20.

12. Irungu, B.N., et al., *Quality assessment of selected co-trimoxazole suspension brands marketed in Nairobi County, Kenya.* 2021. **16**(9): p. e0257625.

13. Ndwigah, S., et al., *The quality of anti-malarial medicines in Embu County, Kenya.* Malar J, 2018. **17**(1): p. 330.

14. Mziray, S., et al., *Quality of selected anti-retroviral medicines: Tanzania Mainland market as a case study.* 2021. **22**(1): p. 46.

15. Bizimana, T., et al., *Investigation of the quality of the 12 most-used antibiotics available in retail private pharmacies in Rwanda.* 2022. **11**(3): p. 329.

16. Seitzer, M., et al., *Quality and composition of Albendazole, Mebendazole and Praziquantel available in Burkina Faso, Côte d'Ivoire, Ghana and Tanzania.* PLoS Negl Trop Dis, 2021. **15**(1): p. e0009038.
